# Supplementary material for: Computationally Discovered Potentiating Role of Glycans on NMDA Receptors
Source: Sci Rep. 2017 Apr 5;7:44578. doi: 10.1038/srep44578 (PMC5381272; doi:10.1038/srep44578)
Supplement: Supplementary Information [file srep44578-s1.pdf]

Computationally Discovered Potentiating Role of Glycans on NMDA Receptors

Anton V. Sinitskiy,[1] *Nathaniel H. Stanley,*[2,3] *David H. Hackos,*[4] *Jesse E. Hanson,*[4] *Benjamin D. Sellers,*[3] *& Vijay S. Pande*[1,5]

[1] Department of Chemistry, Stanford University, Stanford, California 94305

[2] Stanford ChEM-H, Stanford University, Stanford, California 94305

[3] Department of Discovery Chemistry, Genentech, Inc., 1 DNA Way, South San Francisco, CA 94080, USA

[4] Department of Neuroscience, Genentech, Inc., 1 DNA Way, South San Francisco, CA 94080, USA

[5] Department of Computer Science and Department of Structural Biology, Stanford University, Stanford, California 94305

Correspondence and requests for materials should be addressed to A.V.S. (email: sinitskiy@stanford.edu) and V.S.P. (email: pande@stanford.edu).

SUPPLEMENTARY INFORMATION

Section S1. Choice of the optimal number of Markov states

The optimal number of Markov states in the Markov state model of the glycosylated GluN1 LBD was chosen by cross-validation with the use of generalized matrix Rayleigh quotient (GMRQ)[1] for the slowest implicit timescale as the score function. 25 various random divisions of the dataset of all MD trajectories for glycosylated GluN1 LBD into a training set (80% of trajectories) and a test set (20% of trajectories) were performed. Markov state models with the number of Markov states ranging from 2 to 1000 were built from each training set, and then the performance of each MSM was scored against the corresponding test set. Scores averaged over 25 various divisions are reported in Fig. S1a. As expected, the average score for training sets increases with the increase of the number of states, while the average score for test sets reaches a maximum value and decreases with the further increase of the number of states because of overfitting. The optimal number of states corresponding to the maximum average test score turns out to be 99. As for the glycosylated GluN2B LBD, a similar procedure leads to the optimum number of Markov states maximizing the average test score to be 118 (Fig. S1b). In both cases, the score is not very sensitive to the number of states in the vicinities of the extrema, allowing us to expect reasonable performance of Markov models in a wide range of the numbers of Markov states, from ~50 to ~500. Taking this lack of sensitivity into account, we used the same number of Markov states, namely 99, to model all four molecular systems under investigation, glycosylated and non-glycosylated GluN1 and GluN2B LBDs, to ensure the comparability of the results for glycosylated and non-glycosylated forms, as well as different subunits.

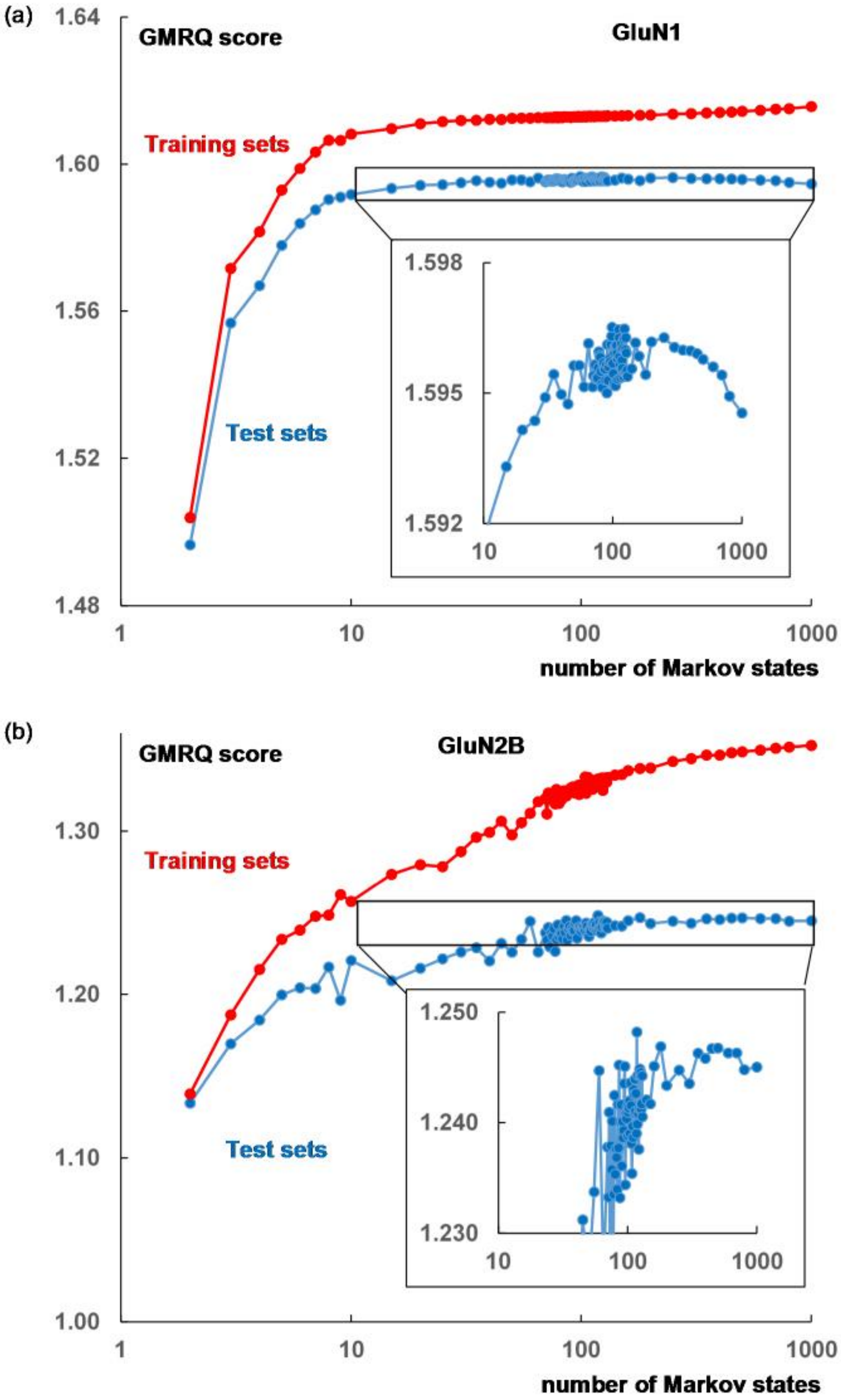

Fig. S1. (a) Cross-validation with the use of generalized matrix Rayleigh quotient (GMRQ) for the slowest implicit timescale as the score function was used to choose the optimal number of Markov states to model glycosylated GluN1 LBD, namely 99. Average scores for training sets (*red*) and test sets (*blue*) over 25 random divisions of the data set into training and test sets are provided. (b) In the case of our glycosylated GluN2B LBD simulations, the optimal number of Markov states appears to be 118, though the model with 99 states is relatively good, too. For comparison, we used Markov models with 99 states both for GluN1 and GluN2B LBDs.

Section S2. Choice of the optimal lag time

Lag time is the distance in time between neighboring frames from MD trajectories used to estimate the MSM transition matrix. Too small values of the lag time can lead to inadequacy of MSM due to a violation of the Markov assumption at short timescales. Too large values of the lag time lead to poorer statistics on transitions. Analysis of the convergence of implicit timescales of MSMs as a function of lag time (Fig. S2) can be used to choose the optimal value of lag time. For our simulations of glycosylated GluN1 LBD, we chose the lag time of 256 ns. In the case of GluN2B LBD, the overall picture is the same as in GluN1 LBD. For the purpose of comparison, this lag time was used to model all four molecular systems under investigation, glycosylated and non-glycosylated GluN1 and GluN2B LBDs.

(see next page)

(a)

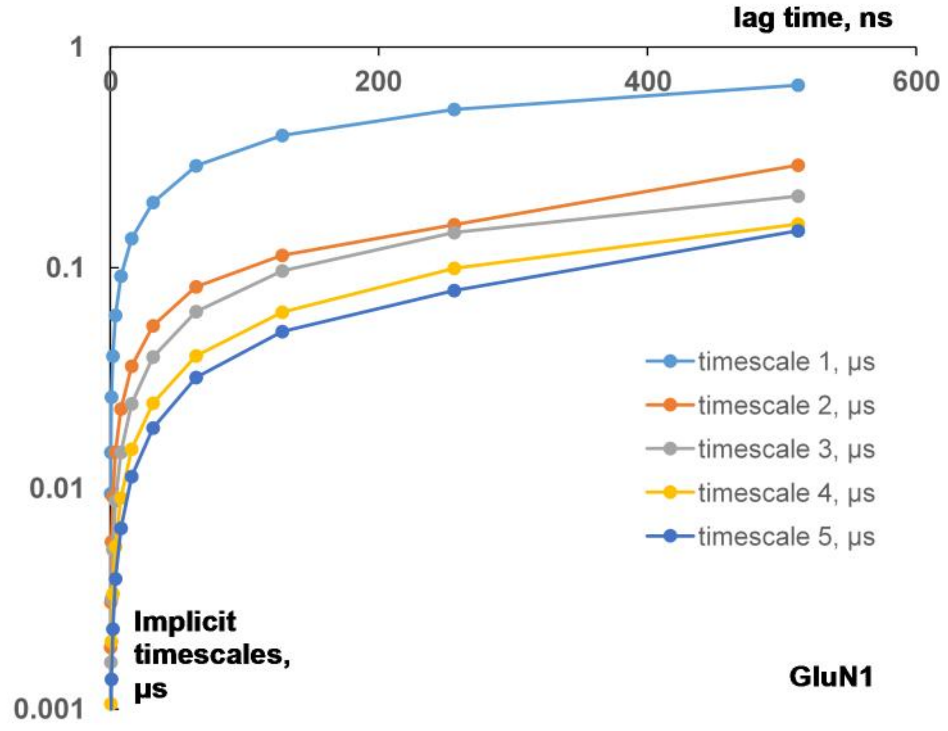

(b)

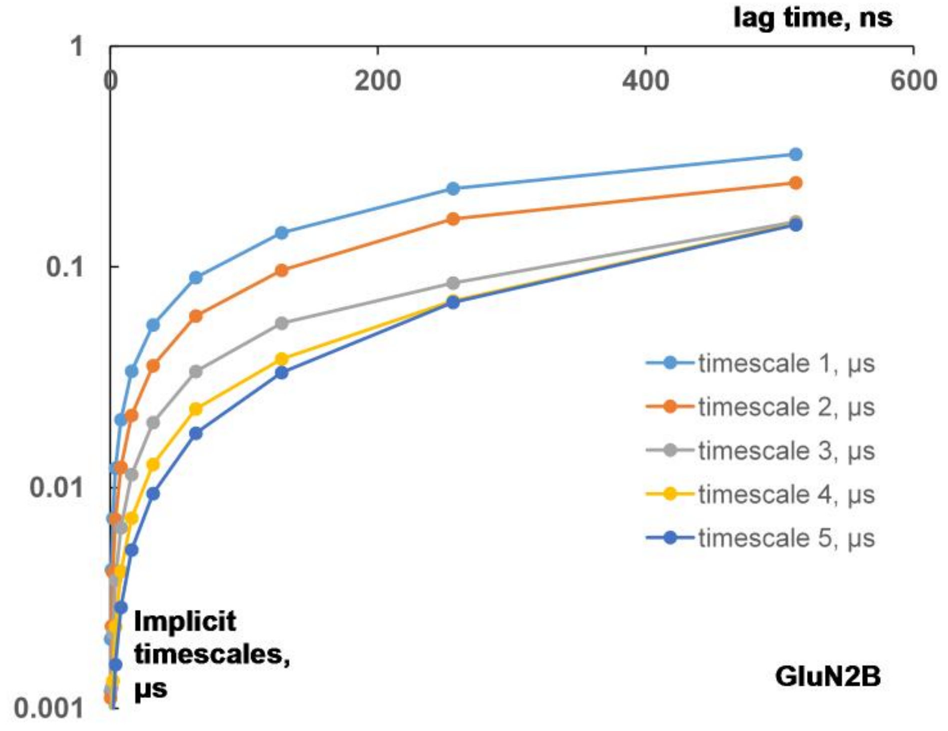

Fig. S2. (a) Five slowest implicit timescales, estimated from MSM for glycosylated GluN1 LBD with 99 states, converge to stationary values as the lag time increases, confirming the

applicability of the Markov assumption. We chose the lag time of 256 ns as a compromise between ensuring Markov and good statistics on transitions between Markov states. Note the gap between the first and the second slowest timescales by a factor of 2.5-4 (depending on the lag time), implying a relatively good decoupling of clamshell opening/closing motion from other conformational transitions in glycosylated GluN1 LBD. (b) In GluN2B LBD, the overall picture with implicit timescales is the same. However, the timescales are faster (up to ~0.2 μs, vs. up to ~0.5 μs in GluN1), and the gap between the first and the second slowest timescales is smaller (a factor of ~1.4-1.6).

Section S3. Estimate of the statistical significance of the difference between probability distribution functions for glycosylated and non-glycosylated GluN1 and GluN2B LBDs by bootstrapping

We performed 1000 rounds of resampling of time series $d(t)$ for glycosylated, and, separately, non-glycosylated GluN1 LBD. For each resampled data set, the probability distribution functions for glycosylated and non-glycosylated GluN1 LBD, denoted below as $f_1(d)$ and $f_2(d)$, respectively, were computed with the use of Markov state models with 99 states and the lag time of 256 ns. Then, the difference between the two probability distributions, $df(d) = f_1(d) – f_2(d)$, was computed for each of 1000 sets of resampled data. Confidence intervals for $df(d)$ were computed at each d by percentile bootstrap of 1000 estimates of $df(d)$. The values of d for which the probability distribution functions for glycosylated GluN1 LBD statistically significantly exceeds the probability distribution functions for non-glycosylated GluN1 LBD were determined as those values of d at which the confidence intervals for $df(d)$ are entirely above zero, and *vice versa*. The confidence intervals for $df(d)$ for the P-values of 95% and 99% are shown in Fig. S3a. Similar computations were performed for GluN2B LBD (Fig. S3b).

(see next page)

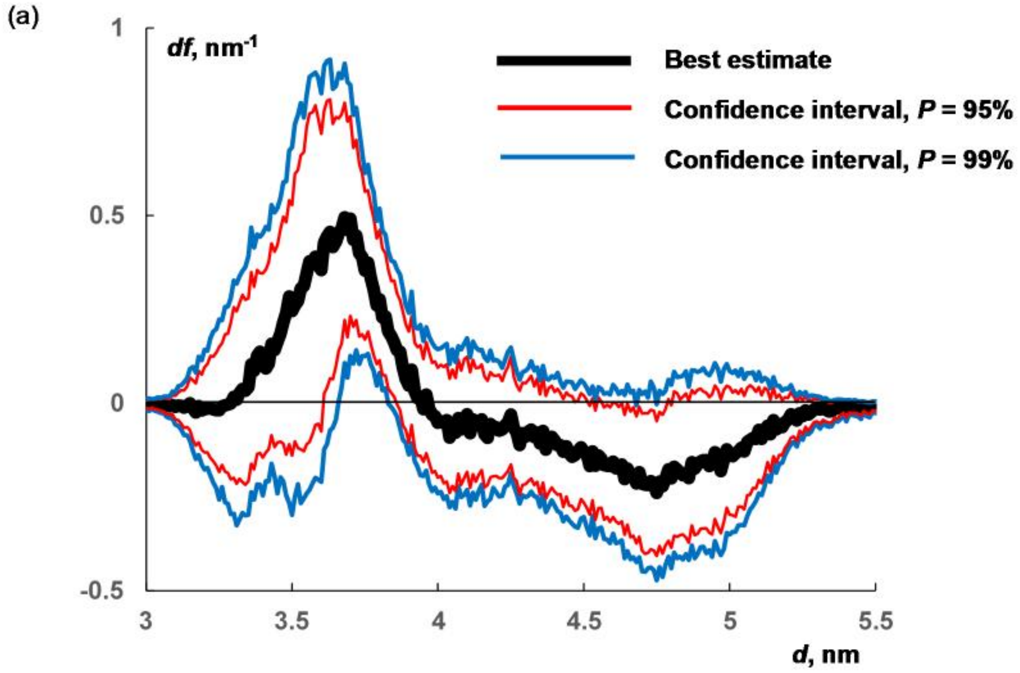

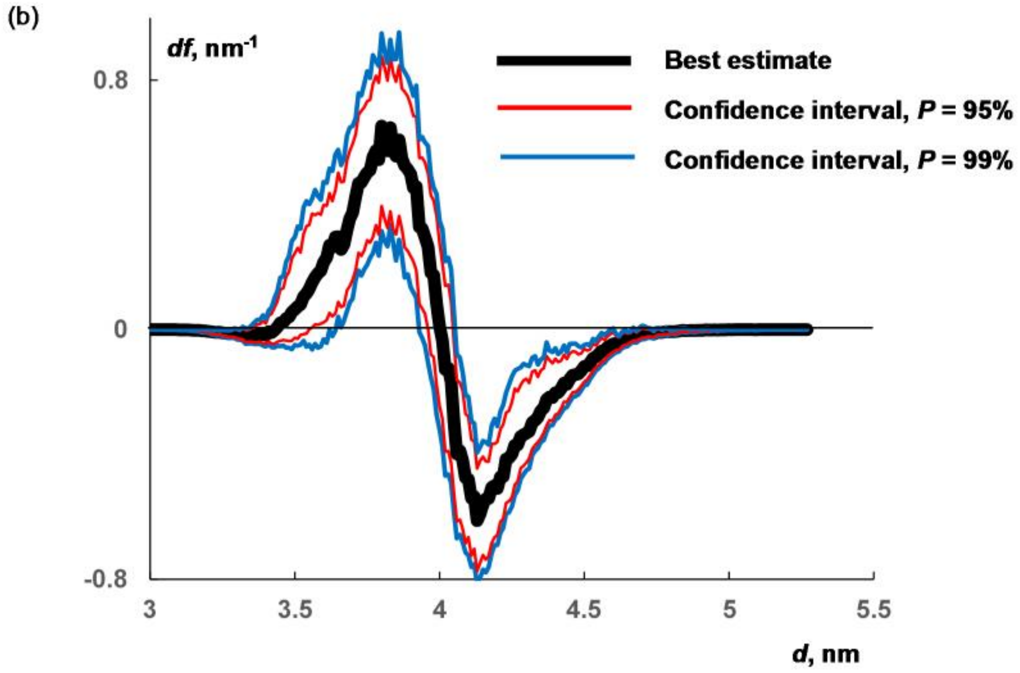

Fig. S3. (a) Conformations of GluN1 LBD with *d* in the range of 3.61 to 3.85 nm are statistically significantly more populated (the confidence interval for *df*, which is the difference between probability distribution functions for glycosylated and non-glycosylated GluN1 LBD, is above zero at these values of *d*), and those with *d* in the range of 4.57 to 4.78 nm are statistically significantly less populated (the confidence interval for *df* is below zero) in glycosylated GluN1 LBD than in non-glycosylated GluN1 LBD (percentile bootstrap, confidence level of 95%).

Black: the best estimate of df based on the available MD trajectories (in other words, the red curve from Fig. 2c minus the blue curve from Fig. 2c). *Red*: the confidence interval for df with the confidence level of 95% estimated by percentile bootstrap. *Blue*: the same, with 99% confidence level. (b) In GluN2B LBD, the glycosylated form is statistically significantly more stable in the ranges of 3.55 to 3.95 nm, and less stable between 4.05 to 4.69 nm (percentile bootstrap, confidence level of 95%).

Section S4. Physical interpretation of slowest timescales of Markov state models

To clarify the meaning of the slowest Markov state model timescale, consider a simplified physico-chemical two-state model of GluN1 or GluN2B LBD, with one open and one closed states (O and C, respectively),

$$O \underset{k_{op}}{\overset{k_{cl}}{\rightleftarrows}} C, \tag{1}$$

and assume that the transitions between these two states are described by the first order reaction kinetics, with the rate constants k_{cl} and k_{op} for closing and opening, respectively. Then the concentration of the open and closed forms will depend on time t as follows:

$$[O] = c_0 + c_1 e^{-(k_{cl}+k_{op})t}, \qquad [C] = c_2 + c_3 e^{-(k_{cl}+k_{op})t}, \tag{2}$$

where constants c_0, c_1, c_2 and c_3 are determined by the initial concentrations of the open and closed forms and the ratio of the rate constants k_{cl} / k_{op}. On the other hand, the time evolution of the ensemble of Markov processes for an n-state Markov chain can be written in comparable notations as

$$[O] = c'_0 + \sum_{i=1}^{n-1} c'_i e^{-t/^2{}_i}, \qquad [C] = c''_0 + \sum_{i=1}^{n-1} c''_i e^{-t/^2{}_i}, \tag{3}$$

where $^2{}_i$ are characteristic timescales of the Markov chain sorted in the descending order, $^2{}_1 \geq {}^2{}_2 \geq \ldots \geq {}^2{}_{n-1}$. If the slowest characteristic timescale $^2{}_1$ is significantly greater than the other characteristic timescales, then for sufficiently large t eq. (2) approximates eq. (3) with

$$^2{}_1 = \frac{1}{k_{cl}+k_{op}} = \frac{\ln 2}{\dfrac{1}{^2{}_{cl}} + \dfrac{1}{^2{}_{op}}}, \tag{4}$$

where $^2{}_{cl}$ is the time over which half of all open conformations would close, provided that closed conformations could not open, and $^2{}_{op}$ is the time over which half of all closed conformations would open, provided that open conformations could not close. The equilibrium concentrations of the open and closed forms of GluN1 or GluN2B LBDs are comparable by the order of magnitude, as Fig. 2(c,e) shows. Therefore $k_{cl} \sim k_{op}$, and hence, $^2{}_{cl} \sim {}^2{}_1$, $^2{}_{op} \sim {}^2{}_1$. Thus, the slowest timescale reported in Table 1 is an aggregate characteristic of complex transitions

between more open and more closed conformations of the GluN1 and GluN2B LBDs. In the first approximation the slowest timescale is comparable, by the order of magnitude, to typical timescales of both opening and closing the clamshell.

Section S5. Methods: more detailed description

Molecular dynamics simulations. Initial 23 geometries of GluN1 LBD were prepared based on all experimental X-ray structures of GluN1 LBD available at the moment when our simulations started (PDB codes: 1PB7, 1PB8, 1PB9, 1PBQ,[2] 1Y1M, 1Y1Z, 1Y20,[3] 2A5T,[4] 4KCC,[5] 4KFQ,[6] 4NF4, 4NF5, 4NF6, 4NF8,[7] 4PE5,[8] 4TLL, 4TLM[9]). The proteins consisted of residues 393 to 546 and 663 to 800 (residue numbering everywhere in this article corresponds to the full GluN1 sequence). The primary structure of GluN1 subunit from *H. sapiens*, isoform 3 (NR1-3), uniprot identifier Q05586-1, was used. The structures were solvated in TIP3P water with sodium and chloride ions in the amount corresponding to the physiological concentration of 0.154 M. Glycosylation was performed with the use of Glycoprotein Builder.[10] Energy minimization, heating and two-stage preequilibration resulted in preequilibrated structures used for production simulations. These preequilibrated structures were within 1 Å from the original X-ray structures in terms of RMSD for the protein backbone atoms.

For the GluN2B simulations, a homology model of the LBD was built from full NMDAR X-ray structures (PDB codes: 4PE5,[8] 4TLL, 4TLM[9]). The six LBD domains (two from each structure) were aligned and used to build a consensus model using Schrodinger's Maestro software (version 2015-1).[11] The template sequence consisted of residues 401 to 604 and 658 to 806, with a glycine linker between 604 and 658. The sequence was based off of the GluN2B subunit from *H. sapiens*, uniprot identifier Q13224-1. The structures were solvated in TIP3P water with sodium and chlorine ions added to neutralize the system. For the glycosylated system, glycosylation was performed with the use of the Glycoprotein Builder.[10]

MD simulations were performed with Amber ff99SB-ILDN[12] and GLYCAM_06i[13] force fields (for the protein and glycan parts, respectively), resulting in 262 MD trajectories for glycosylated and 196 MD trajectories for non-glycosylated GluN1 LBD, with the aggregate duration of 107 and 106 µs, respectively. For the GluN2B LBD, 247 trajectories for the glycosylated and 613 trajectories for the glycosylated form were performed, resulting in an aggregate simulation time of 86 and 344 µs, respectively. Simulations were run on Folding@home and various types of GPUs available on Stanford computer clusters (Sherlock, XStream). Depending on the type of the used GPU, some of the trajectories were generated in OpenMM with hydrogen reweighting, constrained length of all covalent bonds and the timestep of 5 fs,[14] and other trajectories were run in Amber with hydrogen reweighting, constrained length of covalent bonds with hydrogen atoms and the timestep of 4 fs.[15] Coordinates of all atoms were recorded every 0.2 ns. The following checks of the resulting trajectories were performed: stability of the volume, potential and total energy of the system; the distance between mirror images of the (glyco)protein created by periodic boundary conditions (in more than 80% of frames exceeded 19 Å, 16 Å, 25 Å and 14 Å for the glycosylated GluN1 LBD, non-glycosylated GluN1 LBD, glycosylated GluN2B LBD and non-glycosylated GluN2B LBD, respectively; in more than 99% of frames, exceeded 15, 11, 16 and 9.5 Å, respectively); RMSD of the protein backbones in neighboring frames in each MD trajectory (always stayed below 4.5 Å for the

timestep of 0.2 ns, and typically equaled ~1.5 Å). Visual molecular dynamics (VMD) package[16] was used to visualize molecular structures.

Interpretation of molecular dynamics trajectories. Markov state models[17] were built to reconstruct the thermodynamic and kinetic properties of the NPT ensembles of glycosylated and non-glycosylated GluN1 LBD and GluN2B LBD proteins at equilibrium from finite-length MD trajectories, each of which did not completely sample the configuration space. For featurization, the distance *d* between C_α atoms in residues 507 and 701 in GluN1 LBD was used to capture the opening/closing motion of the module. This choice of the residues followed that used in the experimental papers on smFRET investigation of GluN1 LBD opening/closing dynamics on the millisecond-to-second timescale.[18,19] In GluN2B LBD, the distance between C_α atoms in residues 503 and 701, which are homologous to residues 507 and 701 in GluN1 subunit, was used. The MSMbuilder 3.3 package[20] was used to construct microstate models with varying number of Markov states. Maximum Likelihood Estimator was used to generate a transition probability matrix T_{ij}, which maps out the probability of transitioning from state *i* at time *t* to state *j* at time $t + {}^{2}$, where ^{2} is the lag time of the model. The optimal number of Markov states was chosen by cross-validation with the use of generalized matrix Rayleigh quotient (GMRQ) for the slowest implicit timescale as the score function (SI, section S1).[1] The Markov lag time was chosen to be 256 ns based on the analysis of the plots for the implied timescales versus lag times used to compute these implied timescales (SI, section S2). MSM-weighted probability distributions were obtained by binning the raw data within each MSM state and weighting it by the MSM equilibrium state population. To estimate the stability of our key conclusions relative to the model framework used to process the MD data, we also present the results for MSMs with a different number of clusters or a different lag time, as well as the timescale estimated from time-structure independent component analysis (tICA),[21] all with the same featurization *d* (Table 1).

References for the Supplementary Information

1 McGibbon, R. T. and Pande, V. S. Variational cross-validation of slow dynamical modes in molecular kinetics. *J Chem Phys* **142**, 124105 (2015).

2 Furukawa, H. and Gouaux, E. Mechanisms of activation, inhibition and specificity: crystal structures of the NMDA receptor NR1 ligand-binding core. *EMBO J* **22**, 2873-2885 (2003).

3 Inanobe, A., Furukawa, H. and Gouaux, E. Mechanism of partial agonist action at the NR1 subunit of NMDA receptors. *Neuron* **47**, 71-84 (2005).

4 Furukawa, H., Singh, S. K., Mancusso, R. and Gouaux, E. Subunit arrangement and function in NMDA receptors. *Nature* **438**, 185-192 (2005).

5 Yao, Y., Belcher, J., Berger, A. J., Mayer, M. L. and Lau, A. Y. Conformational analysis of NMDA receptor GluN1, GluN2, and GluN3 ligand-binding domains reveals subtype-specific characteristics. *Structure* **21**, 1788-1799 (2013).

6 Kvist, T., Steffensen, T. B., Greenwood, J. R., Mehrzad Tabrizi, F., Hansen, K. B., Gajhede, M., Pickering, D. S., Traynelis, S. F., Kastrup, J. S. and Brauner-Osborne, H. Crystal structure and pharmacological characterization of a novel N-methyl-D-aspartate

(NMDA) receptor antagonist at the GluN1 glycine binding site. *J Biol Chem* **288**, 33124-33135 (2013).

7 Jespersen, A., Tajima, N., Fernandez-Cuervo, G., Garnier-Amblard, E. C. and Furukawa, H. Structural insights into competitive antagonism in NMDA receptors. *Neuron* **81**, 366-378 (2014).

8 Karakas, E. and Furukawa, H. Crystal structure of a heterotetrameric NMDA receptor ion channel. *Science* **344**, 992-997 (2014).

9 Lee, C. H., Lu, W., Michel, J. C., Goehring, A., Du, J., Song, X. and Gouaux, E. NMDA receptor structures reveal subunit arrangement and pore architecture. *Nature* **511**, 191-197 (2014).

10 Woods, R. J. *Glycoprotein Builder*, <http://glycam.org/tools/molecular-dynamics/glycoprotein-builder/upload-pdb>

11 Schrödinger Release 2015-1: Maestro, version 10.1, Schrödinger, LLC, New York, NY, 2015.

12 Lindorff-Larsen, K., Piana, S., Palmo, K., Maragakis, P., Klepeis, J. L., Dror, R. O. and Shaw, D. E. Improved side-chain torsion potentials for the Amber ff99SB protein force field. *Proteins* **78**, 1950-1958 (2010).

13 Kirschner, K. N., Yongye, A. B., Tschampel, S. M., Gonzalez-Outeirino, J., Daniels, C. R., Foley, B. L. and Woods, R. J. GLYCAM06: a generalizable biomolecular force field. Carbohydrates. *J Comput Chem* **29**, 622-655 (2008).

14 Eastman, P., Friedrichs, M. S., Chodera, J. D., Radmer, R. J., Bruns, C. M., Ku, J. P., Beauchamp, K. A., Lane, T. J., Wang, L. P., Shukla, D., Tye, T., Houston, M., Stich, T., Klein, C., Shirts, M. R. and Pande, V. S. OpenMM 4: A Reusable, Extensible, Hardware Independent Library for High Performance Molecular Simulation. *J Chem Theory Comput* **9**, 461-469 (2013).

15 Hopkins, C. W., Le Grand, S., Walker, R. C. and Roitberg, A. E. Long-Time-Step Molecular Dynamics through Hydrogen Mass Repartitioning. *J Chem Theory Comput* **11**, 1864-1874 (2015).

16 Humphrey, W., Dalke, A. and Schulten, K. VMD: visual molecular dynamics. *J Mol Graph* **14**, 33-38, 27-38 (1996).

17 Pande, V. S., Beauchamp, K. and Bowman, G. R. Everything you wanted to know about Markov State Models but were afraid to ask. *Methods* **52**, 99-105 (2010).

18 Dolino, D. M., Cooper, D., Ramaswamy, S., Jaurich, H., Landes, C. F. and Jayaraman, V. Structural dynamics of the glycine-binding domain of the N-methyl-D-aspartate receptor. *J Biol Chem* **290**, 797-804 (2015).

19 Cooper, D. R., Dolino, D. M., Jaurich, H., Shuang, B., Ramaswamy, S., Nurik, C. E., Chen, J., Jayaraman, V. and Landes, C. F. Conformational transitions in the glycine-bound GluN1 NMDA receptor LBD via single-molecule FRET. *Biophys J* **109**, 66-75 (2015).

20 Beauchamp, K. A., Bowman, G. R., Lane, T. J., Maibaum, L., Haque, I. S. and Pande, V. S. MSMBuilder2: Modeling Conformational Dynamics at the Picosecond to Millisecond Scale. *J Chem Theory Comput* **7**, 3412-3419 (2011).

21 Schwantes, C. R. and Pande, V. S. Improvements in Markov State Model Construction Reveal Many Non-Native Interactions in the Folding of NTL9. *J Chem Theory Comput* **9**, 2000-2009 (2013).
